# Tanshinones: Sources, Pharmacokinetics and Anti-Cancer Activities

**DOI:** 10.3390/ijms131013621

**Published:** 2012-10-22

**Authors:** Yong Zhang, Peixin Jiang, Min Ye, Sung-Hoon Kim, Cheng Jiang, Junxuan Lü

**Affiliations:** 1 Department of Biomedical Sciences and Cancer Biology Center, Texas Tech University Health Sciences Center School of Pharmacy, 1300 S. Coulter, Amarillo, TX 79106, USA; E-Mails: yong.zhang@ttuhsc.edu (Y.Z.); peixin.jiang@ttuhsc.edu (P.J.); 2 State Key Laboratory of Natural and Biomimetic Drugs, School of Pharmaceutical Sciences, Peking University, Beijing 100191, China; E-Mail: yemin@bjmu.edu.cn; 3 Cancer Preventive Material Development Research Center, College of Oriental Medicine, Kyunghee University, Seoul 130-701, Korea; E-Mail: sungkim7@khu.ac.kr

**Keywords:** tanshinones, sources, pharmacokinetic, anti-cancer

## Abstract

Tanshinones are a class of abietane diterpene compound isolated from *Salvia miltiorrhiza* (Danshen or Tanshen in Chinese), a well-known herb in Traditional Chinese Medicine (TCM). Since they were first identified in the 1930s, more than 40 lipophilic tanshinones and structurally related compounds have been isolated from Danshen. In recent decades, numerous studies have been conducted to investigate the isolation, identification, synthesis and pharmacology of tanshinones. In addition to the well-studied cardiovascular activities, tanshinones have been investigated more recently for their anti-cancer activities *in vitro* and *in vivo*. In this review, we update the herbal and alternative sources of tanshinones, and the pharmacokinetics of selected tanshinones. We discuss anti-cancer properties and identify critical issues for future research. Whereas previous studies have suggested anti-cancer potential of tanshinones affecting multiple cellular processes and molecular targets in cell culture models, data from *in vivo* potency assessment experiments in preclinical models vary greatly due to lack of uniformity of solvent vehicles and routes of administration. Chemical modifications and novel formulations had been made to address the poor oral bioavailability of tanshinones. So far, human clinical trials have been far from ideal in their design and execution for the purpose of supporting an anti-cancer indication of tanshinones.

## 1. Introduction

Danshen or Tanshen, the dried root or rhizomes of *Salvia miltiorrhiza* Bunge, has been used in Traditional Chinese Medicine (TCM) in China and many Asian countries as preventive or therapeutic remedies for coronary heart diseases, vascular diseases, stroke, hyperlipidemia, endangiitis, arthritis and hepatitis [1,2]. Fufang Danshen, a composite multi-herbal TCM formula containing Danshen as the major ingredient, is officially listed in the *Chinese Pharmacopoeia* for many indications. Fufang Danshen Dripping Pill (one of the commercial forms of Fufang Danshen) has completed Phase II clinical trials for evaluating the efficacy and safety in patients with chronic stable angina pectoris in the USA (No. NCT00797953).

**Figure 1:**
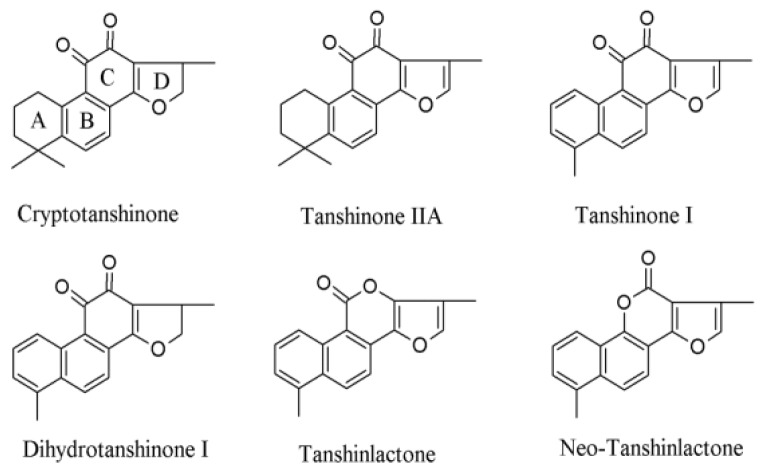
Chemical structures of major tanshinones and tanshinlactones.

Since the 1930s when tanshinones were first isolated from Danshen by Nakao *et al.* [3], more than 90 chemical constituents have been identified. They can be classified into two major groups: more than 40 lipophilic constituents [2,4] and more than 50 hydrophilic compounds, respectively [1]. Tanshinones are a class of lipophilic abietane diterpene compounds, including cryptotanshinone (CT), tanshinone IIA (TIIA), tanshinone I (TI), dihydrotanshinone I (DH-TI) (Figure 1), isotanshinone I, tanshinone IIB, methyltanshinone, isocryptotanshinone I, isocryptotanshinone II, *etc*. [1,4–9]. More recently identified structurally-related tanshinlactone and neo-tanshinlactone (Figure 1) have been reported to be more potent for anti-cancer activities [2]. Hydrophilic constituents from Danshen include danshensu (also known as salvianic acid A or salvianic acid B), protocatechuic acid, protocatechuic aldehyde, rosmarinic acid, salvianolic acid A, salvianolic acid B, and salvianolic acid C (the last three also known as lithospermic acids A–C, or magnesium lithospermates A–C, or tanshinoates A–C), *etc*. [1,5,10,11]. In addition to these two major classes of constituents, baicalin, 5,3′-dihydroxy-7,4′-dimethoxy flavanone, ursolic acid, β-sitosterol, daucosterol, vitamin E and tannin have been identified from Danshen [12]. Numerous studies reported the isolation, identification, synthesis, bioactivities, pharmacokinetics and pharmacology of Danshen constituents. Tanshinones and hydrophilic constituents from Danshen have been extensively investigated for the cardiovascular activities [13–16].

The chemistry and biosynthesis as well as total chemical synthesis of tanshinones have been thoroughly reviewed by Lee and colleagues from the Natural Products Research Laboratories, University of North Carolina, Chapel Hill, North Carolina [2,4]. The anti-cancer properties of tanshinones or tanshinone-containing herbal formula have been investigated more recently. TIIA and CT are the two most extensively studied in this respect, followed by TI and DH-TI. These tanshinones exhibit broad anti-cancer properties in cell culture models, which had been discussed to some extent in several reviews [2,4,17], but pharmacokinetics of tanshinones have not been systematically reviewed.

In this article, we review the natural and alternative sources of tanshinones and their pharmacokinetic characteristics. We provide an in-depth analysis of anti-cancer activities *in vitro* and in preclinical animal cancer models. We update information on cancer-related clinical studies of TIIA and tanshinone-containing TCM formulas. The databases PubMed, SciFinder and CAJViewer (full text articles in Chinese) were used to search literatures covering 1930s–July 2012.

## 2. Sources of Tanshinones, Preparative and Analytical Methodologies

### 2.1. Isolation, Purification and Analytical Methodologies

Tanshinones were first isolated in 1934 from the intensely red rhizomes (roots) of *S. miltiorrhiza* [3]. Their isolation from this and other *Salvia* species usually involved conventional methanolic extraction (MeOH) method. The crude extract was subjected to silica gel column chromatography, using CH_2_Cl_2_-MeOH mixture as elution solvent. Each fraction was re-chromatographed to enhance the concentration by using a gradient of C_6_H_6_-MeOH mixture as the mobile phase. Pigments from each fraction were further purified through recrystallization and preparative thin-layer chromatography [18–20]. Thin-layer chromatography (TLC) was reported as a rapid, sensitive, and accurate method in quantitative determination of both aqueous and lipophilic compounds of *S. miltiorrhiza* and could be employed in quality control of Danshen production [21–23].

High-performance liquid chromatography (HPLC) has been used to simultaneously determine both the aqueous phenolic and non-polar diterpenoid constituents of Danshen products with diode array (DAD) and/or evaporative light scattering (ESL) detectors [10,24,25]. HPLC coupled with electrospray ionization quadrupole ion trap mass spectrometry (HPLC/ESI-IT-MS) or electrospray ionization time-of-flight mass spectrometry (HPLC/ESI-TOF-MS) has been used to provide complementary information for HPLC-DAD by differentiating the isotopic components in Danshen products [9,26]. More recent reports of ultra-high-performance liquid chromatography coupled with quadrupole time-of-flight mass spectrometry (UHPLC/qTOF-MS) showed better qualitative and quantitative analyses of diterpenoids from 12 *Salvia* species regarding resolution, sensitivity, reproducibility and structural information to differentiate positional isomers [27].

Countercurrent chromatography (CCC) is a liquid-liquid partition chromatographic technique with a support-free liquid stationary phase [28–30]. Compared with the classical liquid chromatography (LC), CCC is advantageous for no loss due to irreversible solute absorption, no surface interference including contamination, surface silanol reaction and deactivation, no restriction to flow speed, and able to handle more sample quantities, making it an ideal technique for the separation and purification of natural products [30]. In 2000, Tian and co-workers reported separation of TI, TIIA and CT through high-speed counter-current chromatography (HSCCC) in a single run by using stepwise elution [31]. Multidimensional HSCCC successfully isolated and purified a set of tanshinone analogs including TI, TIIA, DH-TI and CT [32]. Similar result was also observed by using HSCCC with two-phase solvent systems composed of *n*-hexane-ethanol-water to obtain relatively pure compounds including CT, TIIA, TI, DH-TI, methylenetanshiquinone and danshenxinkun B in a stepwise elution mode [33]. In a recent study, DH-TI, 1,2,15,16-tetrahydrotanshiquinone, CT, TI, TIIA, neo-przewaquinone A and miltirone were obtained in an one-step separation by HSCCC with light petroleum–ethyl acetate–methanol–water as the two phase solvent system [34]. Furthermore, a gradient elution model has been developed for CCC systems and was successfully used to separate DH-TI, CT, TI, TIIA and 1,2-dihydrotanshinquinone with a biphasic liquid system composed of hexane-ethyl acetate-ethanol-water [35]. Additionally, chemical fingerprints of *S. miltiorrhiza* were developed to identify major active components by using HSCCC [7,36].

In recent years, the development and application of molecularly imprinted polymers (MIPs) have attracted increasing interest [37]. MIP is a synthetic polymer with molecular recognition sites, which is prepared using molecular imprinting approach. Molecular imprinting involves arranging polymerizable functional monomer around a template through non-covalent, reversible covalent interactions, or metal ion mediated interactions, forming cross-linked polymer matrix through copolymerization, and subsequent removal of the template [38–40]. MIPs are robust, highly selective, workable in organic solvent, low cost, and could be casted in bulk, making it an attractive candidate in natural product extraction. In a recent study, an MIPs system has been established using core-shell structural nanoparticles and optimized by Doehlet design and computational modeling [41]. It exhibited large capacity, high recognition selectivity and fast kinetics in the separation of tanshinone IIA from crude extracts of *S. miltiorrhiza*. The recovery yield of tanshinone IIA reached 93% with a one-step extraction and its purity was more than 98% [41].

### 2.2 Biosynthesis of Tanshinones in *S. miltiorrhiza*

Terpenes or terpenoids in higher plants are synthesized through at least two distinct pathways, the mevalonic acid (MVA) pathway occurring in the cytosol and the non-MVA or 2-*C*-methyl-d-erythritol-4-phosphate (MEP) pathway occurring in the plastids [42]. Tanshinones are generally considered to be synthesized via the MEP pathways, which involve and generate the monoterpenes, certain sesquiterpenes, diterpenes, carotenoids, and the side chains of chlorophylls and plastoquinone. The MVA pathway is responsible for the synthesis of sterols, certain sesquiterpenes, and the side chain of ubiquinone [43–45]. However, the MVA pathway might also play a role in the synthesis of tanshinones for the extensive crosstalk between the two pathways has been reported [46,47].

**Figure 2:**
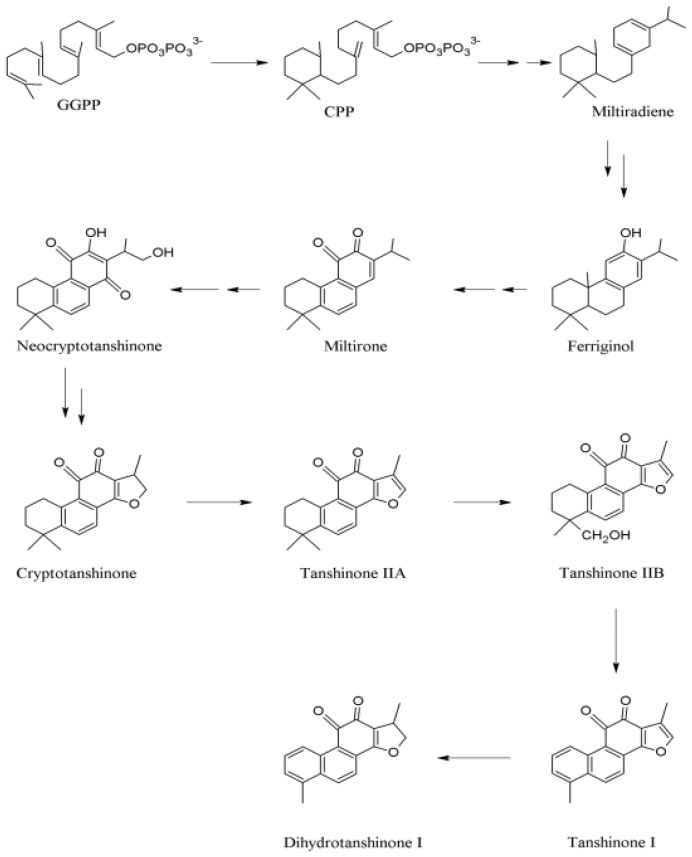
Proposed biosynthetic pathways of tanshinones in *Salvia miltiorrhiza* [48]. Modified based on Wang & Wu 2010. By permission of Springer.

In MEP pathways, 1-deoxy-d-xylulose-5-phosphate synthase (DXS) and 1-deoxy-d-xylulose-5- phosphate reductoisomerase (DXR) have been identified as the first two enzymes [49,50]. DXS catalyzed pyruvate and glyceraldehyde-3-phosphate (GA-3P) into 1-deoxy-d-xylulose-5-phosphate (DXP), which is consecutively converted into MEP through the action of DXR. Then MEP undergoes a series of conversion and results in the formation of isopentenyldiphosphate (IPP) and dimethylallyldiphosphate (DMAPP), the universal precursors of more than 30,000 terpenes [33,51]. The next step after the synthesis of IPP and DMAPP is chain elongation. By reacting with carbonium ion, IPP leads to the production of geranyldiphosphate (GPP). GPP, with an active allylic phosphate group, would reversely react with IPP to produce farnesyl pyrophosphate (FPP) [52]. Then, geranylgeranyldiphosphate synthase (GGPPS) catalyzes the consecutive condensation of three IPP molecules with DMAPP to give a C_20_ compound, geranylgeranyldiphosphate (GGPP), an essential linear precursor for the biosynthesis of diterpenes [53] (Figure 2).

GGPP could be transformed into labdedienyl/copalyldiphosphate (CPP) through CPP synthase, followed by the conversion of CPP to miltiradiene catalyzed by CPP-specific class I diterpene synthase termed as kaurene synthase-like (KSL) [54]. Wang and Wu proposed a hypothetical biosynthetic pathway in which miltiradiene was a key intermediate (Figure 2) [48]. Aromatization and hydroxylation, due to the unstable cyclohexan-1,4-dienes on C ring of miltiradiene, led to ferruginol, which sequentially produced miltirone and neocryptotanshinone by the installation of other groups. Neocryptotanshinone would stepwisely convert into CT, TIIA, TIIB and TI, and probably DH-TI (Figure 2).

### 2.3 Botanical and Alternative Sources of Tanshinones

To date, more than 40 tanshinones or structurally related compounds have been identified from *S. miltiorrhiza*. Many of them have also been identified from related *Salvia* species, which include *S. argentea*, *S. aerea*, *S. bowleyana*, *S. bulleyana*, *S. campanulata*, *S. castanea*, *S. columbariae*, *S. constanea*, *S.* deserta, *S. dabieshanensis*, *S. evansiana*, *S. flava*, *S. honania*, *S. digitaloides*, *S. kiaometiensis*, *S. kiangsisensis*, *S. meiliensis*, *S. paramiltiorhiza*, *S. plectranthoides*, *S. sinica*, *S. trijuga*, *S. prionitis*, *S. przewalskii*, *S. vasta*, and *S. yunnanensis* [55–66]. Although *S. miltiorrhiza* was scribed the only official source of Danshen, 17 other *Salvia* species, such as *S. przewalskii*, *S. yunnanensis*, *S. bowleyana* and *S. trijuga*, have been collected, traded, and used in China herbal markets as substitutes [67,68].

*S. miltiorrhiza* grows in the hilly area of China, Mongolia, Korea, and Japan. Because wild *S. miltiorrhiza* became in short supply due to rapidly growing demanding and over-harvesting, it has been extensively cultivated since the 1970s [69]. Currently, the source of commercial Danshen mainly depends on the field-cultivated *S. miltiorrhiza* plants [70]. However, the cultivated herbal materials have disadvantages including long maturation time (over 20 months), cultivar degeneration, yield reduction by herbivores and pathogens, and quality issues with pesticide residues or heavy metals [71]. Consequently, alternative sources of tanshinones are sought to meet growing pharmaceutical demand. These, in general, involve plant cell/organ cultures. Table 1 summarizes their strengths and intended products.

## 3. Pharmacokinetics of Tanshinones

### 3.1. The Pharmacokinetic (PK) Characteristics of Single Administered Agents or as Mixtures

The major tanshinones (TIIA, CT, TI, DH-TI) have been studied in a number of animal models including rats, rabbits and pigs. Table 2 summarizes representative studies and maximal/peak concentration (*C*_max_) in plasma (central) compartment, providing useful reference ranges to evaluate cell culture based studies and guidance on dosing regimens for future efficacy studies. In spite of differences in the analytical methods, dosing solvents/vehicles, and lack of uniformity in reporting of PK parameters and data interpretation, some general conclusions could be reached.

First, tanshinones are poorly bioavailable with conventional delivery formulations through oral administration (*p.o.*). This was best illustrated by a systematic comparison of the area under the curve (AUC) of *p.o.* intraperitoneal (*i.p.*), *vs*. intravenous (*i.v.*) administration of CT by Zhang and colleagues [94] (See Table 2). The *p.o.* and i.p. bioavailability in rats was estimated as 2.1% and 10.6%, respectively, when CT was dosed at 100 milligrams per kilogram (mg/kg) body weight. Maximal/peak concentration (*C*_max_) values in the nano-molar (nM) to lower micromolar (μM) range after *p.o.* administration were reported in most studies on tanshinones, except that Qiao *et al.* [95] reported for TIIA a *C*_max_ value that was several orders of magnitude higher than most reports (see Table 2). The low aqueous solubility and poor membrane permeability might be responsible for impeding the absorption of TIIA [96]. Zhang and coworkers have shown [94] in rats that a mechanism for the poor intestinal absorption of CT is the presence of an efflux pump (p-glycoprotein) on intestinal apical lumen cells to pump CT into the luminal side.

Second, the drug-concentration time courses of tanshinones fit well into two-compartment or multiple-compartment models after *i.v.* administration in rats, rabbits and pigs. There is usually a very fast component with a half-life of under 10 min (Table 2) and a slower component with half-life ranging 1–3 h. Pharmacokinetic interactions can occur between tanshinones and polyphenolic extracts of *Salvia miltiorrhiza* Bunge after intravenous administration, as seen in the study of Guo *et al.* [97] in rats by PK of the marker compound TIIA and salvianolic acid B (Sal B) as a marker for polyphenolic extract. The authors documented an increase of AUC of about 2-fold in both the low and medium dose groups (2 and 4 mg/kg) for TIIA and Sal B, while there was at least a 14- and 5-fold significant increase (*p* < 0.01) for TIIA and Sal B in the high dose groups, respectively, which was due to a significant (*p* < 0.01) reduction in total plasma clearance. The peak plasma concentrations (*C*_5min_) of TIIA were significantly increased by including the polyphenolic extract in the emulsion (up to 28-fold) (Table 2). However, such interactions did not appear to be as dramatic when examined by oral delivery as shown in the study of Yang *et al.* [107], although some shifts in the absorption of TIIA and its transfer from central compartment to the peripheral compartment were noted.

Third, *in vivo* metabolism or biotransformation of some tanshinones, especially CT to TIIA, has been noted in a number of species (pig, rat) [100–102]. For example, CT is likely biotransformed into TIIA after absorption, a process that might be enhanced by the other active components of Danshen extract [100]. New analytical methods with LC/MS/MS hyphenated technologies for simultaneously detecting multiple tanshinones and their metabolites are leading to greater insights on the issue.

At the absorption level, based on *in situ* intestine perfusion (i.s.i.p.), the diffusion of TIIA and CT in rats would plateau when their concentrations reached a certain level, suggesting that the absorption of TIIA and CT *in vivo* would behave in a manner like active transport or facilitated diffusion [108]. A number of factors including pH and efflux drug pumps could affect absorption of CT [94]. In a study with the small intestine of rats, the complex components in Danshen extract could significantly affect the absorption of the TIIA and CT [109]. Additionally, it has been reported that other constitutes in Danshen extract could improve the distribution and elimination of CT, extend its mean residence time, and increase its bioavailability [99]. Some studies have investigated the PK behaviors of tanshinones in compound formulas for herb-herb interactions. After *i.v.* administration of a phenolic extract of *S. miltinorrhiza* with a tanshinone-rich extract, the plasma retention of TIIA was significantly increased [97] (Table 2).

In summary, systemic concentrations of tanshinones after *i.p.* or oral administration were found in the nano-gram per milliliter (ng/mL) range, that is, nM to sub-μM. Therefore, interpretation of cell culture based studies should take this information into consideration to judge the likelihood of mechanistic relevance.

### 3.2. Structural Modification of Tanshinones

Tanshinones are hard to make into a solution for injection due to their high hydrophobicity and are poorly absorbed when given orally or intraperitoneally. The reported absolute oral bioavailability for CT was only about 2.1% in rats [94] (Table 2). Structural modifications have been made to address the solubility problem and improve intestinal absorption of tanshinones.

**Figure 3:**
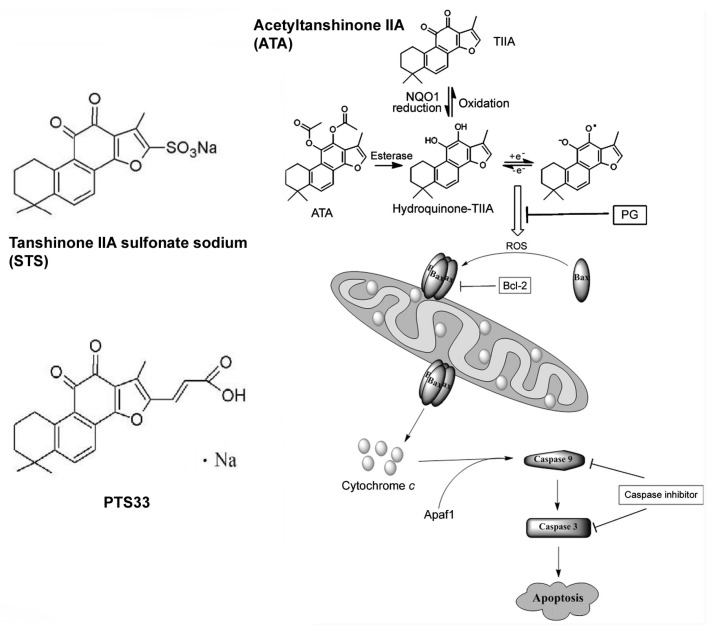
Structures of tanshinone derivatives with increased water solubility. ATA and its likely metabolism [116]. By permission of Elsevier Limited.

Sodium tanshinone IIA sulfonate (STS) is one of water-soluble derivatives of TIIA (see Figure 3 for structure). In China, STS has been widely used to treat patients with cardiovascular diseases for more than 30 years [110,111]. Clinically, STS is effective for coronary artery disease and angina pectoris, likely through its antioxidant activities [112]. Additionally, recent pharmacology research suggested that STS possessed a broad range of pharmacological functions including protecting the myocardium by attenuating hypertrophy, immune-mediated liver injury via modulating NF-κB and IFN-γ/STAT1 pathways, and exhibiting a strong vasodilating effect against vasoconstriction by activating conductance Ca^2+^-sensitive K^+^ channels [113–115].

### 3.3. Improving Pharmacokinetics through Novel Formulations

Solid lipid nanoparticles (SLN) have been considered as promising carriers to achieve sufficient bioavailability of poorly water-soluble drugs [117]. SLN can protect pharmaceutical agents from adverse external conditions and offer better control over the drug release profile [20]. TIIA-loaded SLN coated with poloxamer 188 extended plasma elimination time and mean residence time of TIIA in rats [118]. It was also observed that TIIA-loaded SLN coated with poloxamer 188 reduced opsonization by serum proteins and macrophage uptake, improving the circulation lifetimes for TIIA in plasma [119]. Additionally, a lipid emulsion of TIIA has been developed and evaluated for its stability, safety and bioactivity [17]. Polylactic acid nanoparticles containing TIIA exhibited better performance in pharmaceutical effects [120]. Furthermore, similar strategy has also been used to increase the oral bioavailability of CT in that a SLN formulation significantly improved the absorption of CT and decreased its metabolism to TIIA [121].

Cyclodextrins (CD) are a family of naturally-occurring, water-soluble cyclic oligosaccharides. They have been widely used in cosmetic, food, chemical, agricultural, and pharmaceutical industries [122]. The functions of CD could be greatly increased by chemically modifying their reactive hydroxyl groups. Solid inclusion complexes of TIIA and TI with β-cyclodextrin (β-CD) could be formed via co-precipitation method [123]. The inclusion interaction between CD and tanshinones satisfied the law of enthalpy-entropy compensation [123].

## 4. Anti-Cancer Activities of Tanshinones

**Figure 4:**
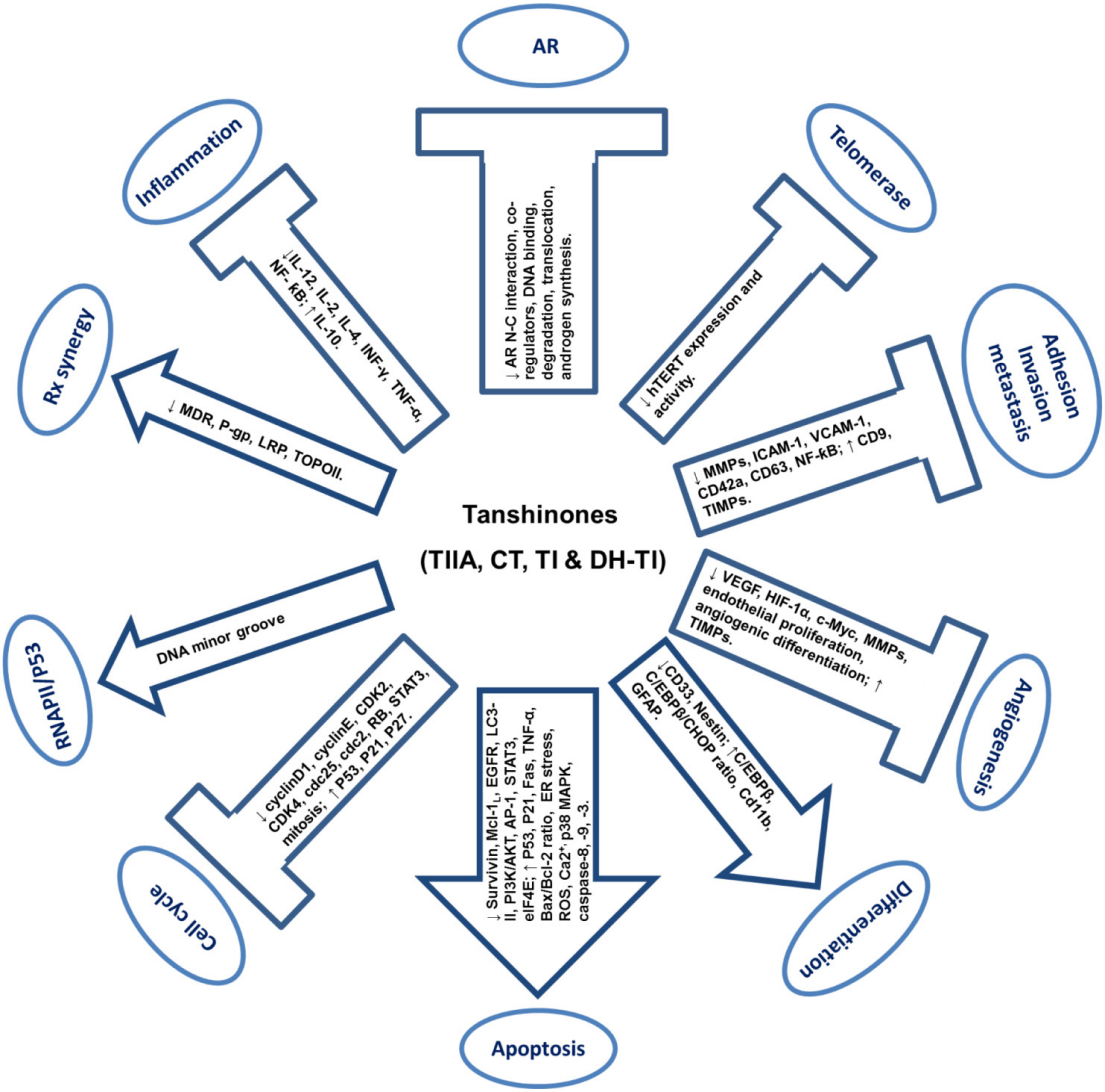
Schematic representations of reported cellular and molecular effects of tanshinones in cell culture models related to cancer. Arrows indicate induction effects. Block T indicates suppression effects. TI: tanshinone I; TIIA: tanshinone IIA; CT: cryptotanshinone; DH-TI: dihydrotanshinone I. ↑: up-regulation or activation; ↓: down-regulation or inactivation.

Tanshinones have been evaluated for anti-cancer properties mostly in cell culture models. The mechanistic relevance should be interpreted, whenever possible, in view of the information on pharmacokinetics discussed in the preceding section. Plasma levels of nM to sub-μM ranges were commonly observed with oral or *i.p.* dosing. Therefore, mechanisms derived from studies with exposure levels of greater than 10 μM of tanshinone compounds should be viewed with caution. Figure 4 provides a schematic summary of the cellular and molecular activities of tanshinones.

### 4.1. Anti-Proliferation and Pro-Apoptosis

#### 4.1.1. Tanshinone I (TI)

TI could induce growth inhibition in various cancer cells including colon [124], stomach [125], breast [126,127], liver [128], prostate [129], lung [130], and leukemia [131] through inhibiting proliferation (induction of cell cycle arrest) and promoting apoptosis, in a broad concentration range from sub-μM to high μM. Up-regulation of p53 and p21 was found to be in association with the induction of cell cycle arrest and apoptosis [124,125]. Increase of Bax/Bcl-2 ratio [124–126,128,131], isruption of mitochondrial membrane potential (MMP) [131] and activation of caspase-3 [124,126,131] might contribute to TI-induced apoptosis, suggesting an involvement of the intrinsic apoptosis pathway. Liu *et al.* [131] reported that TI-induced apoptosis was associated with inhibition of PI3K/AKT kinases in K562 and HL-60 leukemia cells, and was mimicked by PI3K inhibitor (LY294002), indicating a possible involvement of PI3K/AKT pathway in TI-induced apoptosis. Moreover, Gong *et al.* [127,129,130] reported that TI inhibited DU 145 prostate cancer, MCF-7 and MDA-MB-231 breast cancer and H1299 lung cancer cell growth in a concentration-dependent manner, in association with down-regulation of Aurora A (a member of the aurora oncogenic family of mitotic serine/threonine kinases over-expressed in cancers). Knock-down of Aurora A significantly attenuated TI-induced cell cycle arrest and apoptosis, suggesting Aurora A as a possible mediator of TI-induced inhibition in these cells. Mechanically, TI decreased Aurora A through epigenetically suppressing acetylation of the histone H3 associated with Aurora A [127]. *In vivo* administration of TI (150 mg/kg and 200 mg/kg of TI in corn oil via oral gavage) retarded the growth of prostate and lung xenograft tumors in immunodeficient mice with minimal side effect [129,130].

#### 4.1.2. Tanshinone IIA (TIIA)

As one of the most extensively investigated tanshinones, TIIA’s anti-neoplastic activities have been reported in various cancer cells including prostate [132,133], breast [134–140], colon [141], lung [142–145], liver [146–154], stomach [155–159], pancreas [160], bile duct [161], kidney [162], ovary [163–165], cervix [166], nasopharynx [167,168], glioma [169], osteosarcoma [170] and leukemia [171–175], in a broad concentration range from sub-micromolar to high micromolar. Sub-apoptotic concentrations of TIIA could induce G_0_/G_1_, S or G_2_/M arrest depending upon the cancer cell types. The induction of cell cycle arrest was associated with up-regulation of P53, P21 and P27, and down-regulation of cyclinD_1_, CDK2, CDK4, cdc25 and cdc2 [132,141,146,147,150,155,156,162]. Higher concentrations of TIIA could induce caspase-dependent apoptosis, which was generally associated with increases of P53, P21, Fas, TNF-α, Bax/Bcl-2 ratio, disruption of mitochondrial membrane potential (MMP), enhancement of cytochrome c release, decreases of survivin, Mcl-1_L_, EGFR and LC3-II, and activation of caspase-8, caspase-9 and caspase-3, suggesting the involvements of both intrinsic (Bax/Bcl-2/MMP/cytochrome c release/caspase-9/caspase3) and extrinsic (Fas/TNF-α/caspase-8/caspase-3) apoptosis pathways [132–137,141–143,146–151,155,162,163,169–171]. TIIA was reported to decrease ErbB-2/HER2/Neu in Colo205 colon cancer xenograft tumors in male severe combined immunodeficient (SCID) mice (20 mg/kg of TIIA in corn oil, oral gavage, once per day for 30 days) [141], but not in MCF-7 and MDA-MD-231 breast cancer xenograft tumors in female nude mice (30 mg/kg of TIIA in Tween 20:ethanol = 1:99, subcutaneous (s.c.) injection, four times per week for four weeks) [137]. The differences in cell types, mouse strains and TIIA treatment solvent vehicles and routes might partially explain the inconsistent results about ErbB-2/HER2/Neu between the two studies. Endoplasmic reticulum (ER) stress-mediated cytotoxicity was reported in TIIA-treated breast cancer BT-20 cells [135]. After exposure to TIIA, ER stress markers including caspase-12 and GADD153 were increased along with decreased Bcl-xL and increased caspase-3 [135]. In addition, increases of reactive oxygen species (ROS) and cellular Ca^2+^ concentration might also be involved in TIIA-induced apoptosis in BT-20 breast cancer cells and A549 and H146 lung cancer cells [142,143].

Interference of mitosis might be another possible mechanism of TIIA to suppress cancer cell proliferation. Zhou *et al.* [176] found that TIIA could arrest cancer cells in mitosis phase (before metaphase-anaphase transition) by disrupting the mitotic spindle, and then subsequently elicited mitochondria-dependent apoptosis. Distinct from the existing therapeutic drugs that cause G_2_/M arrest through interfering with the microtubule structure (such as vincristine or taxol), TIIA destroyed the mitotic spindle in mitotic cells rather than the microtubule structure in interphase cells, suggesting a preference of TIIA to kill mitotic cells over interphase cells. Moreover, the *in vivo* pro-apoptosis activity of TIIA was also established in xenograft tumors including colon (20 mg/kg of TIIA in corn oil, oral) [141] and breast (30 mg/kg of TIIA in Tween 20:ethanol = 1:99, s.c. injection or 30 mg/kg of TIIA, *i.p.* injection, respectively) [137,138].

Several signal transduction pathways including PI3K/AKT, p38 MAPK and AP-1 pathways might be involved in the inhibitory action of TIIA. Won *et al.* [133] reported that TIIA treatment reduced the expression of PI3K p85 subunit and phosphorylation of AKT and mTOR in a concentration-dependent manner. Moreover, combination of TIIA with a PI3K inhibitor (LY294002) enhanced TIIA-induced apoptosis in LNCaP and PC-3 prostate cancer cells which contained high AKT, but did not in MDA-MB-231 breast cancer cells which contained undetectable AKT, suggesting a possible involvement of PI3K/AKT survival pathway [133]. Jiao *et al.* [163] found that TIIA treatment activated pro-apoptosis p38 MAPK pathway and then suppressed the expression of pro-survival gene survivin, as well as drug-resistance genes excision repair cross-complementing gene 1 (ERCC1) and lung resistance protein (LRP) in a time- and concentration-dependent manner. Blockade of p38 MAPK pathway by its inhibitor SB203580 attenuated TIIA-induced inhibition of these pro-survival genes and apoptosis, suggesting a mediator role of p38 MAPK. In addition, TIIA was used as an AP-1 inhibitor to suppress AP-1-mediated transcription of aldo-keto reductase 1B10 (AKR1B10), an oncoprotein over-expressed in human liver and lung tumors [177].

#### 4.1.3. Cryptotanshinone (CT)

Anti-proliferation and pro-apoptosis activities of CT have been reported in multiple cancer cells including cervical cancer, cholangiocarcinoma, melanoma, rhabdomyosarcoma and leukemia [178–182]. CT-induced cell cycle arrest and apoptosis were in association with up-regulation of P53 and P21, and decreases of cyclin A_1_, cyclin B_1_, cdc2 and survivin [178–180]. CT at 2.5–40 μM inhibited the growth of human Rh30 rhabdomyosarcoma and DU 145 prostate cancer cells in a concentration-dependent manner, in association with suppression of mTOR pathway, decreased cyclin D_1_ expression and RB phosphorylation. Constitutive activation of mTOR attenuated CT-induced decrease of cyclin D_1_ expression and RB phosphorylation as well as cell growth inhibition, suggesting mTOR pathway as an important target [181]. Similarly, CT at 5–20 μM induced cell cycle arrest and apoptosis in drug-resistant leukemia K562/ADM cells, in association with down-regulation of cyclin D_1_ and Bcl-2. The inhibition of cyclin D_1_ and Bcl-2 happened on post-transcriptional level accompanied by inhibiting eukaryotic initiation factor 4E (eIF4E), in that over-expression of eIF4E attenuated the inhibitory effect of CT [182].

Through screening more than 3000 herbal products, Shin *et al.* [183] identified CT as a selective inhibitor of STAT3, a member of the signal transducer and activator of transcription (STAT) family [184]. Overexpression and constitutive activation of STAT3 promote tumorigenesis by transcriptionally up-regulating its down-stream targets such as cyclin D_1_, survivin and VEGF, which are involved in proliferation, apoptosis and angiogenesis [185–188]. CT at 7 μM inhibited DU145 prostate cancer cell (STAT3 highly active) growth accompanied by inhibition of STAT3 Tyr705 phosphorylation and the expression of down-stream targets. Mechanism investigation showed that CT suppressed STAT3 dimerization, nuclear translocation and DNA binding [183]. Compared with CT, TIIA at the same concentration range did not inhibit STAT3 activity in this study [183]. However, a previous study reported that TIIA at 3.4–27 μM could inhibit the constitutive activation of STAT3 in rat C6 glioma cells [189]. The different cell types (human prostate cancer *vs.* rat glioma) might be a possible explanation about the discrepancy between the two cell culture studies. Since CT converts to TIIA *in vivo*, the anti-cancer efficacy in animal models are likely the combined effect of CT with TIIA.

In addition, Park *et al.* [190] reported that CT at 1–10 μM sensitized DU 145 cells to Fas-mediated apoptosis through inhibiting Fas-mediated Bcl-2 expression. Many cancer cells express death-receptor Fas on their membranes, however, some are resistant to Fas-mediated apoptosis because activation of Fas can simultaneously up-regulate the anti-apoptosis protein Bcl-2 in these cells through JNK and p38 MAPK kinase pathways. Co-treatment with CT significantly blocked Fas-induced activation of these kinases and consequently inhibited Fas-induced up-regulation of Bcl-2, and then sensitized Fas-mediated apoptosis in these cells.

#### 4.1.4. Dihydrotanshinone I (DH-TI)

Though the studies on DH-TI are not as many as other major tanshinones, DH-TI has been reported to exert the strongest potency and unique mechanisms on some tumor cells. Shi *et al.* [191] evaluated the potency of four major tanshinones (TI, TIIA, CT and DH-TI) on SPC-A-1 lung cancer cells, and found DH-TI was the most active, followed by TI, TIIA and CT. Similarly, DH-TI was the most potent out of the four tanshinones to inhibit HepG2 liver cancer cell growth. Interestingly, though all the four major tanshinones could induce ROS in HepG2 cells, DH-TI was the only one that triggered reactive oxygen species (ROS)-mediated apoptosis through activating p38MAPK pathway, suggesting some unique signaling by DH-TI [192]. DH-TI also demonstrated stronger inhibitory effect on MCF-7 and MDA-MB-231 breast cancer cells than the same concentration of TI and CT. DH-TI at sub- and low μM levels (0.4–2 μM) significantly induced G_1_ arrest and caspase-mediated apoptosis in these cells, in association with reduction of cyclin D_1_, cyclin D_3_, cyclin E, CDK4 and Bcl-xL expression, increase of P27 expression and cytochrome c release, inactivation of CDK2 and CDK4, and activation of caspase-9, caspase-7 and caspase-3 [193]. Moreover, *in vivo* administration of DH-TI (10 mg/kg of DH-TI in DMSO, *i.p.* injection, three times per week for four weeks) retarded MDA-MB-231 xenograft tumors in nude mice [193]. DH-TI at sub- or low micromolar levels (0.4–5.4 μM) could induce apoptosis in DU 145 prostate cancer cells via inducing classical endoplasmic reticulum (ER) stress, evidenced by up-regulation of ER stress markers such as GRP78 and GADD153, eIF2α, JNK and XBP1 mRNA splicers. DH-TI treatment caused significant accumulation of polyubiquitinated proteins and HIF-1α, indicating DH-TI as a potential proteasome inhibitor, which was known to induce ER stress or enhance ER stress-mediated apoptosis [194]. Moreover, co-treatment with an ER stress inhibitor (salubrinal) attenuated DH-TI-induced apoptosis, supporting a mediator role of ER stress in DH-TI-induced apoptosis [194].

Taken together, these studies demonstrated the anti-proliferation and pro-apoptosis activities of tanshinones on various cancer cells, involving multiple targets. It is hard to judge how likely these targets and mechanisms are invoked *in vivo* at the present because most of the reported studies did not have *in vivo* data to corroborate the pharmacodynamic targets.

### 4.2. Pro-Differentiation of Cancer Cells

Aside from anti-proliferation and pro-apoptosis, induction of differentiation is another important mechanism to suppress cancer, as de-differentiation is a hallmark of cancer. Pharmacological induction of terminal differentiation has already been used to treat leukemia with all-trans retinoic acid (ATRA, a pro-differentiation drug) and arsenic-base agents [195–197].

Induction of differentiation by TIIA has been documented in several studies. In 1997, Yuan *et al.* [198] investigated the differentiation effect of TIIA on leukemia cells, and found that after 4 days of TIIA treatment (1.7 μM), more than half of HL-60 human leukemia cells were induced into mature neutrophils, in which metamyelocytes were 46%, and banded and segmented neutrophils were 12%, respectively. The HL-60 cell growth was inhibited, accompanied by G_0_/G_1_ arrest, decreased c-Myc and increased c-Fos. Similar results were obtained from later studies [199–201]. Liang *et al.* [199] reported that after five days of TIIA treatment (1.7 μM), the majority of leukemia NB4 cells (91.3%) converted into more differentiated cells, including 26% of myelocytes and metamyelocytes as well as 68% of band form and segmented neutrophils. The induced expression of membrane cluster differentiation (CD) antigens was consistent with the differentiation fates, *i.e.*, CD33 (a marker of myeloid cells) was decreased and CD11b (a marker of terminal differentiation) was increased. ATRA-resistant leukemia cells could also respond to TIIA. After 4 days of incubation with 3.4 μM of TIIA, ATRA-resistant leukemia MR-2 cells underwent morphological and functional differentiation as indicated by small cell bulk, decreased nucleus/cytoplasm ratio, rough chromatin, disappearance of nucleolus, formation of anomalous nucleus, appearance of metamyelocyte, decrease of CD33 and increase of CD11b, and reduction of nitroblue tetrazolium test (NBT reduction, a marker of functional differentiation) [202]. Using acute promyelocytic leukemia (APL) cells isolated from patients, Liang *et al.* [203] tested the differentiation effect of TIIA on primary leukemia cells. After incubation with 1.7 μM of TIIA for seven days, morphological and functional differentiation was observed, accompanied by G_0_/G_1_ arrest and growth inhibition. In addition, a synergistic effect of TIIA and ATRA (1.7 μM of TIIA plus 0.4, 0.8 or 1.6 μM of ATRA, respectively) on cell differentiation was reported in leukemia NB4 cells. The growth inhibition rate, differentiation rate, NBT reduction, differentiation markers CD33 (decrease) and CD11b (increase) were much higher in the combination group than ATRA or TIIA alone [204]. Moreover, Zhang *et al.* [173] found that C/EBPβ/CHOP ratio might be a determinant of TIIA-induced differentiation response of leukemia cells. C/EBPβ and CHOP (also known as GADD153) are members of the C/EBP family of leucine zipper transcription factors which interact with specific DNA sequences and affect the transcription of proliferation- and differentiation-related genes [205]. Expression of C/EBPβ drives immature granulocytes to enter into terminal differentiation [206], yet expression of CHOP reverses this differentiation direction through inhibiting C/EBPβ binding to its target DNA [207]. TIIA at 0.34–3.4 μM induced leukemia NB4 and MR-2 cell differentiation as indicated by increased expression of CD11b, which was associated with up-regulation of C/EBPβ expression and increased C/EBPβ/CHOP ratio [173]. However, higher concentration of TIIA (34 μM) showed less pro-differentiation effect, along with up-regulation of CHOP expression but a decrease of C/EBPβ/CHOP ratio. Very likely the onset of apoptosis subverted the differentiation program at the higher exposure concentration. The data suggested that the expression and ratio of C/EBPβ and CHOP might play important roles in mediating sub-apoptotic range of TIIA-induced differentiation in these leukemia cells [173].

TIIA-induced differentiation was also reported in cancer cells of solid organs. Wang *et al.* reported that TIIA at 0.3 μM induced human U251 glioma cell differentiation as indicated by an increase of GFAP (a marker of differentiated astrocyte) and a decrease of nestin (a marker of undifferentiated astrocytic precursors) [169,208,209]. Similarly, morphological differentiation was observed in cervical and osteosarcoma tumor cells exposed to TIIA treatment [210,211]. Taken together, these studies suggest TIIA as a potential differentiation inducer of tumor cells, especially leukemia.

### 4.3. Inhibition of Angiogenesis

Angiogenesis is a process of new vessel formation from existing ones and plays a crucial role in tumor growth and metastasis. Endothelial cells can migrate toward the angiogenesis stimuli released from tumor cells and proliferate and differentiate to form a new vessel [212]. Neo-angiogenesis not only supplies the necessary nutrients and oxygen [213], but also allows tumor cells to escape into the circulation and metastasize to distant sites [214]. Recently studies suggest that tanshinones can suppress angiogenesis through inhibiting endothelial proliferation and angiogenic differentiation, in association with modulation of angiogenic regulators including but not limited to VEGF, HIF-1α, c-Myc, matrix metalloproteinases (MMPs) and tissue inhibitors of metalloproteinases (TIMPs).

In 2005, tanshinones were reported to inhibit the proliferation of fetal bovine serum (FBS)-induced proliferation of cultured rat vascular smooth muscle cells (VSMCs) in a concentration-dependent manner, in association with inactivation of ERK1/2, increased P21 and down-regulated cyclin D_1_ abundance [215]. Later, Tsai *et al.* [216] used human umbilical vein endothelial cells (HUVECs) as a model and found that TIIA at 1, 5, 10 and 20 μM concentration-dependently inhibited FBS-induced migration, TNF-α-induced invasion, and extracellular matrix (ECM)-induced tube formation. They also showed TIIA suppressed phorbol-12-myristate-13-acetate (PMA)-induced angiogenesis in chicken embryo chorioallantoic membrane (CAM) assay. The anti-angiogenesis activity of TIIA was associated with regulating MMP and TIMP secretion from vascular endothelial cells, *i.e.*, pro-angiogenesis MMP-2 secretion was decreased but anti-angiogenesis TIMP-2 secretion was increased. This study also indicated that the anti-angiogenesis action of TIIA might be independent of the cytotoxic effect, because there was no sign of apoptosis or necrosis when HUVECs were treated with up to 20 μM of TIIA [216].

The anti-angiogenesis activity of TIIA was also evaluated using cancer cell models. TIIA at low micromolar concentrations could inhibit the 2-dimension (2-D) and 3-dimension (3-D) migration of MDA-MB-435 cells (now a confirmed melanoma cell line), measured by wound-healing and transwell migration assays, respectively [217,218]. TIIA also inhibited the tube formation of newborn cattle aortic endothelial cells (NCAECs) stimulated by co-incubation with MDA-MB-435 cells [217]. TIIA-induced inhibition of angiogenesis was accompanied by down-regulation of pro-angiogenesis factors such as VEGF, HIF-1α and c-Myc [217,218]. Similarly, TIIA-induced inhibition of VEGF, HIF-1α and c-Myc was also reported in other cancer cells [219–223]. Moreover, an *in vivo* study showed that TIIA (0.5, 1 and 2 mg/kg, *i.v.* injection) decreased serum VEGF levels and microvessel density (MVD) in C26 colon cancer xenografts in mice, in a dose-dependent manner [223].

In addition to TIIA, other tanshinones also possess potential anti-angiogenesis activities. Gong *et al.* [129] reported that TI suppressed HUVECs growth with IC_50_ of ~2.5 μM and inhibited HUVECs migration and tube formation, in concentration-dependent manner. *In vivo* administration of TI (150 and 200 mg/kg of TI in corn oil, oral gavage) decreased MVD in DU 145 prostate cancer and H1299 lung cancer xenograft tumors [129,130]. Nizamutdinova *et al.* [110] reported that, in a concentration range from 1–50 μM, TI inhibited TNF-α-induced production of VEGF from HUVECs and MDA-MB-231 breast cancer cells, as well as VEGF-stimulated tube formation of HUVECs. CT was reported to suppress cell growth and bFGF-induced invasion and tube formation of bovine aortic endothelial cells (BAECs) in a concentration-dependent manner [224]. DH-TI at low micromolar levels suppressed HUVECs migration, invasion and tube formation in a concentration-dependent manner [225], and inhibited HIF-1α expression and HIF-1α-mediated transcription [226]. CT and DH-TI (0.1 and 0.2 μg/egg) inhibited angiogenesis in CAM assay as indicated by decreased MVD, in a dose-dependent manner [225,227].

Taken together, these reports suggest tanshinones as potential inhibitors of angiogenesis via inhibiting proliferation and angiogenic differentiation of endothelial cells as well as by targeting angiogenic stimuli from cancer cells and perhaps other cells from the cancer microenvironment.

### 4.4. Inhibition of Adhesion, Migration, Invasion and Metastasis

Cancer metastasis is a complex process through coordination between cancer cells and surrounding microenvironment, including breaking through the basement membrane barriers, intravasation into vessel, survival in the circulation, extravasation from vessels, and survival and proliferation in the target tissues. Adhesion, migration and invasion are indispensable steps for cancer metastasis, therefore are important pharmacological targets of novel anti-cancer agents.

In 1999, Zhang *et al.* [228] investigated the effect of several natural products including TIIA on the expression of membrane cluster differentiation (CD) antigens of human PGCL3 pulmonary giant cell carcinoma cells. They found that CD42a and CD63 might promote but CD9 might inhibit PGCL3 cell invasion through Matrigel. TIIA treatment (9, 34 and 68 μM) decreased CD42a and CD63 expression but increased CD9, indicating a possible inhibition of TIIA on the invasion capability of PGCL3 cells [228]. Later, Liu *et al.* [229] found that TIIA at 10–50 μM inhibited leukemia NB4 cell adhesion to extracellular matrix (ECM) component (laminin, fibronectin, collagen and vitronection)-coated surfaces and invasion through Matrigel in a concentration-dependently manner. Shan *et al.* [230] reported that TIIA at low μM concentrations (3.4–6.8 μM) inhibited migration and invasion of human HT29 and SW480 colon cancer cells in a concentration- and time-dependent manner. TIIA decreased urokinase plasminogen activator (uPA), matrix metalloproteinase (MMP)-2 and MMP-9, increased tissue inhibitors of metalloproteinase (TIMP)-1 and TIMP-2, and suppressed nuclear accumulation of NF-κB P65, a crucial subunit of nuclear factor-kappaB (NF-κB) pathway. TIIA also inhibited liver metastasis of colon cancer in a mouse tail vein metastasis model. After 5 weeks of TIIA treatment (20 and 80 mg/kg, daily intragastric administration), the liver metastasis rate of SW480 colon cancer cells was decreased by 40% and 61%, respectively [230]. A similar concentration range of TIIA also inhibited *in vitro* migration and invasion and *in vivo* metastasis rate of human HepG2 and SMMC-7721 liver cancer cells in a concentration- and time-dependent manner, in association with inhibition of NF-κB pathway as well as MMP-2 and MMP-9 expression and activity [231]. TIIA-induced inhibition of MMP expression and activity was also observed in other cancer cells such as osteosarcoma and stomach tumor [170,232].

Nizamutdinova *et al.* [110] reported that TI suppressed MDA-MB-231 breast cancer cell metastasis involving modulating surface adhesion molecules. TI at 5–50 μM completely inhibited the expression of intercellular adhesion molecule-1 (ICAM-1) and vascular cell adhesion molecule-1 (VCAM-1) in TNF-α-stimulated HUVECs, as well as the adhesion of MDA-MB-231 cells to HUVECs and migration of MDA-MB-231 cells through extracellular matrix. Knockdown of ICAM-1 and VCAM-1 by siRNA mimicked TI-induced inhibition of MDA-MB-231 cell adhesion to HUVECs. Moreover, *in vivo* administration of TI (10 mg/kg, daily *i.p.* injection for 4 weeks) reduced lung metastasis of MDA-MB-231 xenograft tumors in nude mice [110]. Similarly, Lee *et al.* [233] reported that TI inhibited migration, invasion and MMPs activity in macrophage conditional medium-stimulated CL1-5 lung cancer cells *in vitro*, and reduced the tumorigenesis and metastasis of CL1-5 lung cancer xenografts in severe combined immunodeficient (SCID) mice, in association with reduction of interleukin-8 (IL-8, an angiogenesis factor promoting angiogenesis and metastasis). Mechanically, the inhibition of IL-8 induced by TIIA likely happened at the transcription level via interfering with the binding of transcriptional factors AP-1 and NF-κB to the IL-8 promoter [233].

### 4.5. Modulation of Inflammatory and Immune Responses

Increasing evidence supports the important role of inflammatory and immune responses for tumorigenesis and therapeutic responses. Several studies have been conducted to evaluate the possible effects of tanshinones on inflammatory and immune responses [115,234–237]. Kang *et al.* [115] investigated the effects of tanshinones on the production of interleukin-12 (IL-12) and interferon-gamma (IFN-γ), cytokines playing important roles in mediating T and natural killer (NK) cell functions. They found that all tested tanshinones (TI, CT and DH-TI) at sub- and low micromalor levels inhibited IL-12 production in lipopolysaccharide (LPS)-activated mouse macrophages in a concentration-dependent manner. DH-TI was more effective than TI and CT. The inhibition of IL-12 appeared to occur at transcriptional level through inhibiting transcriptional factor NF-κB to bind to the IL-12 promoter. Similarly, TIIA decreased IFN-γ production in keyhole limpet hemocyanin (KLH)-primed mouse lymph node cells [115]. Later, Qin *et al.* [234] investigated the protection of TIIA on immune-mediated liver injury and found that TIIA could significantly attenuate concanavalin A (ConA)-induced immune-mediated liver injury in mice, as indicated by decreases of plasma alanine aminotransferase (ALT) and aspartate amino transferase (AST) levels. TIIA increased anti-inflammatory cytokine IL-10 and reduced pro-inflammatory cytokines IL-2, IL-4, IFN-γ and tumor necrosis factor alpha (TNF-α) [234]. These results were generally consistent with previous reports that tanshinones or tanshinone-containing agents possessed anti-inflammatory properties [235–237]. Taken together, these limited data suggest a possible role of tanshinones in regulating inflammatory and immune responses.

### 4.6. Inhibition of Telomerase

Telomerase is a ribonucleoprotein complex that synthesizes the repetitive G-rich DNA at each 3′-end of the chromosome [238]. Telomerase can promote carcinogenesis and progression via maintaining telomeric DNA length to overcome replicative senescence and inhibiting apoptosis by mechanisms independent of telomeric DNA synthesis [239,240], and promote proliferation via cross-talking with other pathways [241]. Telomerase is up-regulated in the majority of cancer cells and is essential for their survival. Telomerase has been an important therapeutic target for developing novel anti-cancer drugs in the past 2 decades.

Several studies reported that TIIA treatment (1.7 μM, 2–6 days) induced apoptosis and differentiation in human HL-60 and K562 leukemia cells, accompanied by inhibition of both the expression and activity of human telomerase reverse transcriptase (hTERT) [242–244]. Similarly, Liu *et al.* [245] reported that TI at 10–50 μM concentration-dependently induced caspase-3-mediated apoptosis, in association with a decrease of hTERT expression and activity in U937, THP-1 and SHI 1 leukemia cells. Soares *et al.* [246] delineated the direct effect of tanshinones on telomerase using an *in vitro* telomerase assembly assay, which allowed identification of small molecules directly affecting telomerase through inhibiting enzymatic activity, blocking specific protein-RNA interaction [247] or suppressing necessary chaperone HSP90 interaction [248,249]. They showed that tanshinones (TI, TIIA and CT) exert direct effect on telomerase, possibly in association with reactive oxygen species (ROS) generation in a test tube [246]. Furthermore, they demonstrated that the ortho-quinone C ring (See Figure 1) of the tanshinones is essential for telomerase inhibition in that replacement of the C rings with a lactone ring caused significant loss of that activity, whereas the aromatic A ring and heteroaromatic D ring seemed dispensable for telomerase inhibition [246].

### 4.7. Interaction with DNA Minor Groove and P53 Activation

Zhang *et al.* [250,251] reported that TIIA could interact with DNA minor groove, and in turn activate P53 and induce apoptosis by interrupting RNA polymerase II (RNAPII)-dependent transcription in cancer cells. It has been documented that groove binding agents (such as distamysin A) can arrest RNAPII-dependent transcription via altering the conformation of the transcribed DNA [252–254]. The transcription arrest will trigger phosphorylation and degradation of RNAPII and in turn activate P53-mediated apoptosis, a process known as RNAPII response [255–257]. TIIA could interact with duplex DNA by binding to the minor groove (preferentially to A/T-rich sequences) [250]. Similar to other DNA groove binders, TIIA treatment induced RNAPII response in H22 liver cancer and K562 leukemia cells [251]. TIIA at 0.4–4 μM increased RNAPII phosphorylation in a concentration-dependent manner. When TIIA concentration rose above 4 μM, RNAPII was fully phosphorylated and began to degrade. P53 was activated by TIIA exactly at the same concentration where the RNAPII level started to be down-regulated. P53 activation and apoptosis were observed in the concentration range of 4–20 μM of TIIA treatments [251]. Moreover, activation of P53 was independent of ataxia-telangiectasia mutated (ATM) activation, suggesting that TIIA-induced P53 activation in these cells was not through ATM-mediated DNA damage-response pathway [251]. Furthermore, *in vivo* administration of TIIA (40 mg/kg of TIIA in 0.5% carboxymethyl cellulose, *i.p.* injection, every other day for 10 days) induced RNAPII down-regulation and apoptosis in H22 xenograft tumors. Taken together, the results provide a possible mechanism of TIIA activating the P53 pathway and inducing apoptosis in some cancer cells.

### 4.8. Modulation of Androgen Receptor Pathway

Recently, we have shown the androgen receptor (AR) pathway as a potential target of tanshinones [258]. The rationale was in part due to Danshen being used to treat acne, an androgen-related dermal lesion [259] and tanshinones’ structural resemblance to androgen. Anti-androgen action was considered as one mechanism of Danshen against acne [260]. A previous study showed that tanshinones could reduce the wet weights of androgen-dependent organs (prostate and seminal vesicle) of male animals [261]. In our study, tanshinones (TI, TIIA and CT) at sub- and low micromolar concentrations suppressed cell growth and AR-dependent transcription in AR-responsive prostate cancer cells in a concentration-dependent manner [258]. TIIA and CT (<2.5 μM) suppressed AR down-stream target prostate specific antigen (PSA) expression without affecting cellular AR level, whereas higher concentrations (>5 μM) showed additional actions on the nuclear translocation and 26S proteasomal-dependent degradation of AR. Compared with TIIA and CT, TI showed less activity on AR pathway [258]. Similar observations were made by others, in that CT and PTS33 (a sodium salt derivative of CT with increased water-solubility for structure) at low concentrations (<2 μM) inhibited AR-dependent transcription without affecting AR protein level; however, at higher concentrations (5–10 μM) promoted AR degradation in LNCaP prostate cancer cells [262–264]. The inhibitory activities of CT and PTS33 at lower concentrations (<2 μM) involved the disruption of AR *N*-*C* terminal interaction, co-regulator recruitment and DNA binding [262–264]. Low concentrations of TIIA may share similar mechanism as low concentrations of CT and PTS33, as far as TIIA at <2.5 μM suppressed AR-dependent transcription without significantly affecting AR abundance [258]. Furthermore, the 4,4-dimethyl group at ring A of TIIA seemed important for the anti-AR activity of TIIA, for the inhibitory activity was reduced when this group was removed or moved to 2- or 3-position at ring A [265]. *In vivo*, TIIA (oral daily, 25 mg/kg, four weeks) decreased AR and PSA levels in LNCaP prostate cancer xenografts, accompanied by a reduction of tumor burden [258]. CT (5 and 25 mg/kg, every other day by *i.p.* for four weeks) decreased tumor weight and the mRNA levels of AR-dependent transactional genes such as PSA and TMPRSS2 in 22Rv1 prostate cancer xenografts, all in dose-dependent manners [262].

It is noteworthy that the mechanisms for tanshinones to inhibit AR transcriptional activity are distinct from classical AR antagonists. Classical AR antagonists including flutamide, bicalutamide and the newly-approved drug enzalutamide (MDV3100) suppress ligand-binding activation of AR via competition with androgen ligands (e.g., testosterone and dihydrotestosterone) to bind the hormone binding pocket within the *C*-terminal ligand binding domain (LBD) of AR. TIIA and CT seemed unlikely to competitively bind the hormone binding pocket, in that the substituting capability of these tanshinones was much lower than androgenic ligand and bicalutamide as revealed by the AR competitor binding assays [258,262]. However, the inhibitory action of tanshinones (TIIA, CT and PTS33) indeed relied on a full-length AR, in that a truncated AR without LBD could not be regulated by tanshinones [262,263,265].

Several reports also indicated that tanshinones might suppress AR signaling via regulating androgen synthesis or metabolites. Li *et al.* [266] reported that CT (27 mg/kg of CT in Tween 80/saline, oral gavage for 14 days) decreased plasma 17α-hydroxy progesterone in male offspring with high circulatory androgen stimulated by prenatal androgenization. Similarly, Yang *et al.* [267] reported that CT (27 mg/kg, 14 day) attenuated the increased plasma androgen level in female offspring receiving prenatal androgenization, in association with reduction of androgen synthesis enzyme CYP17. Zhao *et al.* [268] reported that CT (600 mg/kg of CT, oral gavage for 8 weeks) reversed the increased plasma testosterone level caused by Akt2 deletion in mice, in association with decrease of CYP17. Furthermore, Qiu *et al.* [269] reported that TIIA (not TI or CT) could activate androgen metabolism enzyme CYP3A-mediated 6β-hydroxylation of testosterone in human liver microsomes, suggesting a potential role of TIIA in regulating androgen metabolism. Yet, Wang *et al.* [270] reported a different result that tanshinones (TI, TIIA, CT and DH-TI) inactivated androgen metabolism enzyme CYP3A2-catalyzed 6β-hydroxylation and CYP2C11-catalyzed 2α-hydroxylation of testosterone in rat liver microsomes. The different species (human *vs.* rat) from which liver microsomes were prepared for the two studies might be a possible explanation for the opposite results (activation *vs*. inactivation). Undoubtedly, more studies are needed to further delineate the effect of tanshinones on androgen metabolism, especially in human body.

Taken together, the studies suggest tanshinones as potential modulators of androgen synthesis and AR signaling pathway. Considering the crucial role of AR pathway in human prostate cancer, these new findings may provide a scientific basis of developing new tanshinone-based therapeutic agents for prostate cancer via suppressing AR pathway.

### 4.9. Synergy with Chemotherapy and Radiotherapy

Tanshinones have also been reported as a sensitizing agent for chemotherapy and radiotherapy. Combination of chemotherapeutic drugs with TIIA achieved significant higher inhibition rate on various cancer cells than the drugs alone. For instance, compared to cisplatin, 5-fluorouracil (5-FU) and oxaliplatin alone, combination of these drugs with TIIA significantly enhanced apoptosis and growth inhibition in HeLa human cervical cancer cells and SMMC-7721 liver cancer cells [271–274]. In an *in vivo* study, He *et al.* [275] reported that combination of cyclophosphamide (CTX, 20 mg/kg) and TIIA (15 mg/kg) achieved significantly better efficacy than CTX alone in a multi-drug resistance (MDR) lung cancer model. The enhanced efficacy was associated with induction of apoptosis and decreases of drug-resistant proteins including p-glycoprotein (P-gp), lung cancer resistance protein (LRP) and topoisomerase II (TOPOII) [275]. Similarly, CT at low μM levels enhanced the potencies of TNF-α, cisplatin, etoposide and 5-FU against cancer cells, in association with induction of endoplasmic reticulum (ER) stress and reactive oxygen species (ROS) production, as well as activation of ERK, JNK and p38 MAPK kinases [276,277]. DH-TI at low micromolar level (3.4 μM) could sensitize the anti-proliferative and pro-apoptotic effects of radiotherapy (2–8 Gy) on Hela cells [278]. Molecular changes associated with the sensitization included decreased expression of HPV E6, cyclin B_1_ and cdc2, and increased expression of P21 and activation of caspase-3. *In vivo* administration of DH-TI (10 mg/kg of DH-TI, i.p. injection for 22 days) significantly reduced tumor volume and weight of irradiated Hela xenografts in mice [278].

## 5. Cancer-Related Clinical Studies

Several clinical reports suggest tanshinone-containing Traditional Chinese Medicine (TCM) formula or TIIA as promising anti-cancer drug candidates (Table 3). The clinical use of TIIA was described in two single-case reports. One case about a 30-year old man diagnosed with acute promyelocytic leukemia (APL) was published in 2006. The patient received all-trans retinoic acid ATRA (20 mg, three times per day) for 14 days, but could not achieve complete remission, then was switched to TIIA (30 mg, oral, twice per day). After 8–12 weeks of TIIA treatment, the patient’s blood (8 weeks) and bone marrow (12 weeks) routines were restored to normal levels, indicating a complete remission [279]. Another case was reported about a 21-year old man with relapsed APL after one year of ATRA, arsenic trioxide, 6-mercaptopurine and methotrexate treatments [280]. After 54 days of TIIA (80 mg, *i.v.* once per day) treatment, the patient reached completely morphological remission without obvious side effects [280]. Well-designed clinical trials with more patients and randomized placebo-controlled design will help to further validate the clinical efficacy of TIIA and other tanshinones.

Multicenter clinical trials in China showed a complete remission (CR) rate of 96.7%–98% and a 5-year overall survival rate of 86.88% were achieved in leukemia patients receiving Realgar-*Indigo naturalis* formula (RIF), a TCM formula containing Realgar, *Indigo naturalis*, Danshen and *Radix psudostellariae* [285–287]. Tetraarsenic tetrasulfide and Indirubin were considered as the major bioactive ingredients of Realgar and *Indigo naturalis*, respectively. The therapeutic benefit of RIF for leukemia patients were also reported by others [288–291]. Following up on the clinical trials, a laboratory study showed that tetraarsenic tetrasulfide and TIIA alone could induce differentiation in leukemia cells, and combination of the three active constituents (tetraarsenic tetrasulfide, TIIA and Indirubin) exerted synergistic effect in both cell culture and murine leukemia models [292].

Compared to chemotherapy alone, Fufang Danshen Injection (injectable formula made from Danshen and other herbs) could significantly attenuate chemotherapy-induced complications and increase the complete remission rate for acute leukemia [281]. Similarly, Fufang Danshen Dripping Pill combined with chemotherapeutic drug gemcitabine and cisplatin increased objective response and clinical benefit response rates than chemotherapy alone (46.3% *vs*. 35.0% and 73.2% *vs*. 50.0%, respectively) [284]. In addition, Fufang Danshen Injection plus chemotherapeutic drug mitomycin and adriamycin through *trans*-umbilical-portal vein perfusion after surgical resection of the primary liver carcinomas significantly delayed the 1- and 2-year recurrence rates compared to the surgical resection alone [282]. Similarly, combination of hepatic artery perfusion of Fufang Danshen Injection with liver transcatheter arterial chemoembolization (TACE) improved the survival and life quality of patients with hepatocellular carcinoma than TACE alone [283].

## 6. Novel Tanshinones and Chemical Modifications

More recently identified novel tanshinone-related compounds by Lee and associates [293–296] have not been studied beyond their initial discovery work. Many of these novel compounds, including neo-tanshinlactone (Figure 1), appear to have greater cytotoxic potency against select cancer cells and better selectivity compared to those reviewed above [297]. Readers should follow up on the research progress dealing with these new entities.

Because of the high hydrophobicity of tanshinones, poor solubility in aqueous medium and poor bioavailability through oral or *i.p.* route of delivery have been the major challenges for pharmaceutical development. Early effort in China with structural modification led to the development of a water-soluble sodium tanshinone IIA sulfonate (STS) (See Figure 3). This TIIA derivative drug has been widely used for patients with cardiovascular disorders in China [298–300]. However, this compound had little apoptosis-inducing ability when tested in six cancer cell lines [301,302]. For this reason, investigators synthesized a novel TIIA compound, acetyltanshinone IIA (ATA) (Figure 3) [116]. ATA exhibited increased water solubility and stronger apoptotic activity on multiple cancer cell lines than TIIA. ATA displayed higher growth inhibition ability on breast cancer especially ErbB-2/HER2/Neu positive cancer cells than normal cells and it inhibited MDA-MB-435 (now a confirmed melanoma cell line) xenograft growth in mice at a dose of 30 mg per kg by *i.p.* injection, three times per week. Mechanistic studies showed that ATA could induce significant reactive oxygen species (ROS) generation, Bax translocation to mitochondria, resulting in mitochondria damage, cytochrome c release, caspase-3 activation and apoptotic cell death (Figure 3). The ATA-induced ROS production and its downstream apoptotic events could be blocked by propyl gallate, an antioxidant agent, indicating the prominent role of ROS in ATA-induced apoptosis. Overexpression of Bcl-2 protein decreased ATA-induced cell death. The authors concluded that ATA was a novel agent with potent *in vitro* and *in vivo* anti-cancer ability and ROS-mediated Bax activation as a mechanism by which ATA induced apoptosis and inhibited cancer cell growth (Figure 3).

Chang and colleagues synthesized a new sodium derivative of CT which they named PTS33 (Figure 3) and tested its effect on androgen receptor (AR) pathway [263]. Their data showed that PTS33 selectively inhibited AR activities, but did not repress the activities of other nuclear receptors, including ERα, GR, and PR. At a concentration of 2 μM PTS33 effectively suppressed the growth of AR-positive prostate cancer (PCa) cells, and had little effect on AR-negative PCa cells. Furthermore, their data indicated that PTS33 could modulate AR transactivation and suppress the AR target genes (PSA, TMPRSS2 and TMEPA1) expression in both androgen responsive LNCaP cells and castration-resistant C4-2 cells. In addition, PTS33 inhibited estrogen/Δ5-androstenediol induced AR activities. Further mechanistic studies indicated that PTS33 inhibited AR function by suppression of AR protein expression, AR N–C interaction and AR co-regulator interaction. The structure of PTS33 could be used as a prototype for development of novel AR signaling inhibitors to treat PCa. So far, the *in vivo* bioavailability and efficacy of PTS33 has not been reported.

## 7. Summary and Perspective

The studies reviewed above suggest that tanshinones possess broad range anti-cancer potential through anti-proliferation, pro-apoptosis, anti-angiogenesis, induction of differentiation, and inhibition of adhesion, migration, invasion and metastasis. Tanshinones may also exert their inhibitory actions through modulating of inflammatory and immune responses, inhibiting telomerase, interacting with DNA minor groove and activating p53 tumor suppressor, or regulating specific pathways such as AR (e.g., TIIA, CT) or STAT3 (e.g., CT). Tanshinones can sensitize cancer cells to apoptosis by current therapeutic drugs. Identification of AR pathway as a target of selected tanshinones provides scientific basis for developing new tanshinone-based agents for prostate cancer in the future.

Cancer-related clinical studies in China suggested potential benefit of tanshinone-containing formulas for cancer patients, yet most of these studies had serious limitations, such as small sample size, absence of necessary control groups, lack of randomization and blinding in trial designs, poorly defined dose-formulation information and content of tanshinones used. Well-designed clinical studies to validate the efficacy of tanshinones in cancer patients await significant efforts to overcome major challenges.

One challenge is the poor water-solubility and bioavailability of natural tanshinones. The chemical modification approaches discussed above have resulted in improved water solubility in all cases and improved anti-cancer efficacy in some instances such as ATA [116], but inactivated apoptosis potency in others such as STS [301,302]. The ongoing pharmaceutical approach through nanoparticle- or lipid-based delivery formulation may help to improve the delivery and bioavailability of tanshinones. Structure-activity relationship (SAR) investigations have produced some insights into the chemical basis of bioactivities of tanshinones. Computational approaches with defined molecular targets (e.g., STAT3 and CT) combined with cell culture assays are necessary to improve the knowledge of molecular targets. Testing the efficacy of defined tanshinone compounds or their combinations in relevant animal cancer models will be a key to pave the way for clinical translational work.

## Figures and Tables

**Table 1:** Alternative sources of *S. miltiorrhiza* and tanshinone production.

Alternative Source	Characteristics	Products	References
Callus culture (root, stem, leaf blade, and petiole)	Well established conventional strategy, potential to form a whole plant, and easy maintenance of tissue in culture	Cryptotanshinone, tanshinone I, and tanshinone IIA	[72–75]
Cell suspension culture	Reliable, sustainable, free of adverse environment factors, automatic control, and scalable for commercial production	Tanshinone I, tanshinone IIA, cryptotanshinone, and ferruginol	[76–79]
Cell immobilization culture	High cell densities, continuous removal of secreted products, reuse of biocatalysts, and protection for shear-sensitive cells by matrix	Tanshinone IIA, cryptotanshinone, and ferruginol	[80,81]
Rhizogenesis	Genetic and biosynthetic stability, plant hormone-independent growth, multi-enzyme biosynthetic potential, and relatively low cost	Tanshinone I, tanshinone IIA, tanshinone IIB, tanshinone V, dihydrotanshinone I, cryptotanshinone, tanshinone VI, and diterpene ferruginol	[82–88]
Crown gall cultures	Fast growth rate, independent of exogenous phytohormones, high productivity of secondary metabolites that are low in normal cell cultures	Cryptotanshinone, tanshinone I, tanshinone IIA, rosmarinic acid, and lithospermic acid B	[89–92]
Endophytic fungi	Economic, reproducible, ecology-friendly, and easy to scale up	Tanshinone I and tanshinone IIA	[93]

**Table 2:** Pharmacokinetic parameters of selected tanshinones (TIIA, CT, TI and DH-TI).

Species	Administered drug/mixture/extracts (marker compound)	Route	Dose mg/kg	PK for	*C*_A+B_, *C*_max_ or C_t_ (μM)	t_1/2β_ (h)	t_1/2γ_ (h)	References
Rat	Danshen extract-lipid emulsion (TIIA)	*i.v.*	2	TIIA	0.144 (*C*_5min_)		2.27	[97]
			4	TIIA	0.20 (*C*_5min_)		2.35	
			8	TIIA	0.932 (*C*_5min_)		2.17	
	Danshen extract-lipid emulsion (TIIA) Plus polyphenolic extract	*i.v.*	2	TIIA	0.81 (*C*_5min_)		3.13	
			4	TIIA	1.17 (*C*_5min_)		3.43	
			8	TIIA	22.78 (*C*_5min_)		4.79	
Rabbit	Danxiongfang formula in Tween80/saline (TIIA)	*i.v.*	2.5	TIIA	10.99 (*C*_A_ + *C*_B_)	0.041	2.25	[98]
	Danxiongfang formula Tween80/saline (CT)	*i.v.*	4.5	CT	15.10 (*C*_A_ + *C*_B_)	0.039	1.42	
Rabbit	CT in Tween80/saline	*i.v.*	4.5	CT	11.89 (*C*_A_ + *C*_B_)	0.036	1.16	[99]
	Danxiongfang formula (CT)	*i.v.*	4.5	CT	15.10 (*C*_A_ + *C*_B_)	0.039	1.42	
Pig	CT in isopropanol solution	*i.v.*	10	CT	10.44 (*C*_A_ + *C*_B_)	0.040	1.08	[100]
				TIIA	2.10 (@*t*_max_ = 4.6 min)		3.15	
		*p.o.*	40	CT	0.15 (@1 h)			
		*i.m.*	20	CT	0.19 (@20 min)			
Rat	CT in aqueous solution	*i.v.*	20	CT	9.57		1.06	[94]
	CT in aqueous solution	*i.p.*	100	CT	2.22 (@*t*_max_ 1.91 h) (10.6% iv AUC)		6.88	
	CT in aqueous solution	*p.o.*	100	CT	0.305 (@*t*_max_ 5.19 h) (2.1% iv AUC)		6.64	
Rat	CT as dispersion	*p.o.*	20	CT	0.085 (@*t*_max_ 4 h)		~4	[101]
				TIIA	0.041 (@*t*_max_ 4 h)		~5	
Rat	CT suspension in 1% Tween80	*p.o.*	5.7	CT	0.037 (@*t*_max_ 0.50 h)	~0.05	~3.9	[102]
				TIIA	0.009 (@*t*_max_ 0.50 h)			
Rat	TIIA	*p.o.*	15	TIIA	**18.9** (@*t*_max_ 0.85 h)	0.55	3.63	[95]
Rat	TIIA in Tween80 suspension	*p.o.*	8	TIIA	0.012 (@*t*_max_ 0.32 h)		3.84	[103]
	CT in Tween80 suspension	*p.o.*	5.7	CT	0.022 (@*t*_max_ 0.56 h)		2.83	
	CT in Tween80 suspension	*p.o.*	5.7	TIIA	0.012 (@*t*_max_ 0.42 h)		3.12	
	Danshen EtOH Extract (TIIA) in Tween80 suspension	*p.o.*	8	TIIA	0.121 (@*t*_max_ 0.64 h)		5.12	
	Danshen EtOH Extract (CT) in Tween80 suspension	*p.o.*	5.7	CT	0.189 (@*t*_max_ 0.58h)		4.80	
Rat	Tanshinones Mixture (TIIA)	*p.o.*	18	TIIA	0.06 (@*t*_max_ 4h)			[104]
	Tanshinones Mixture (CT)	*p.o.*	18	CT	0.027 (@*t*_max_ 4 h)			
Rat	Tanshinones Mixture (TIIA)	*p.o.*	4.1	TIIA	0.009 (@*t*_max_ 0.54 h)		2.07	[105]
	Tanshinones Mixture (CT)	*p.o.*	1.91	CT	0.002 (@*t*_max_ 0.42 h)		1.13	
	Tanshinones Mixture (TI)	*p.o.*	1.1	TI	0.006 (@*t*_max_ 0.42 h)		3.00	
	Tanshinones Mixture (DH-TI)	*p.o.*	1.91	DH-TI	0.012 (@t_max_ 0.79 h)		1.69	
Rat	Tanshinones Mixture (TIIA)	*p.o.*	5.79	TIIA	0.076(@*t*_max_ 0.61 h)	0.40	3.70	[106]
	Tanshinones Mixture (CT)	*p.o.*	9.82	CT	0.145 (@*t*_max_ 0.86 h)	0.69	2.81	
	Tanshinones Mixture (TI)	*p.o.*	3.9	TI	0.198 (@*t*_max_ 0.60 h)	0.94	4.72	
	Tanshinones Mixture (DH-TI)	*p.o.*	3.58	DH-TI	0.041(@*t*_max_ 0.74 h)	0.54	3.65	
Rat	Danshen tanshinone extract (TIIA) in CMC 0.5%	*p.o.*	20	TIIA	0.057 (@*t*_max_ 6.67 h)		7.04	[107]
	tanshinone extract + salvianolic acid B extract	*p.o.*	20	TIIA	0.060 (@*t*_max_ 4.35 h)		5.86	
	tanshinone extract + notoginseng extract	*p.o.*	20	TIIA	0.054 (@*t*_max_ 4.33 h)		6.90	
	Tanshinone extract + borneol (Bingpian)	*p.o.*	20	TIIA	0.066 (@*t*_max_ 2.00 h)	0.032	6.28	
	All extracts combined	*p.o.*	20	TIIA	0.075 (@*t*_max_ 3.67 h)	0.041	6.02	

*t*_1/2β_: initial phase elimination half-life; *t*_1/2γ_: terminal elimination phase half-life; C_A_ + C_B_: Extrapolated initial concentrations of two compartments; *C*_max_: maximal/peak concentration; *i.v. i.p. i.m.* and *p.o*.: intravenous, intraperitoneal, intramuscular and oral administration, respectively.

**Table 3:** Cancer-related clinical studies of tanshinone IIA (TIIA) and tanshinone-containing traditional Chinese medicine (TCM) formulas.

Tanshinone tested/Tanshinone containing formula	Cancers	Treatment(s)	Number of patients	Clinical benefit	References
TIIA	Leukemia	TIIA (80 mg, *i.v.* once per day)	Single case report	CR	[280]
TIIA	Leukemia	TIIA (30 mg, *p.o.*, twice per day)	Single case report	CR	[279]
Fufang Danshen Injection	Leukemia	Control: chemotherapy only; Treatment: chemotherapy plus Fufang Danshen Injection (20–30 mL, *i.v.*, once per day)	Control: 46 Treatment: 86	Fufang Danshen slightly increased CR rate, but significantly attenuate the side effects of chemotherapy.	[281]
Fufang Danshen Injection	Liver carcinoma	Control: surgical resection only; Treatment: surgical resection plus chemotherapy and Fufang Danshen Injection (250 mL, TUV perfusion, once per day for 7 days, repeat every 3–4 week )	Control: 30 Treatment: 30	1- and 2-year recurrence rates (control *vs.* treatment): 60.7% *vs.* 15.3% (*p* < 0.05) and 75.1% *vs.* 30.0% (*p* < 0.05).	[282]
Fufang Danshen Injection	Liver carcinoma	Control: TACE only; Treatment: TACE plus Fufang Danshen Injection (16 mL, *i.v.* once per day for 7 days)	Control: 37 Treatment: 53	1-, 2- and 3-year survival rate (control *vs.* treatment): 72.97% *vs*. 79.25%, 43.24% *vs*. 66.04% (*p* < 0.05) and 24.32% *vs*. 45.28% (*p* < 0.05).	[283]
Fufang Danshen Dripping Pill	Pancreatic carcinoma	Control: chemotherapy only; Treatment: chemotherapy plus Fufang Danshen Dripping Pill (250 mg, *p.o.* 3 times per day)	Control: 40 Treatment: 41	CR + PR and CR + PR + SD rates (control *vs*. treatment): 35.0% *vs*. 46.3% and 50.0% *vs*. 73.2% (*p* < 0.05).	[284]
RIF (formula)	Leukemia	Control: ATRA (30 mg per day) plus placebo for RIF. Treatment: RIF (2.25–7.5 g per day, *p.o.*) plus placebo for ATRA	Control: 59 (placebo controlled) Treatment: 61	CR rate (control *vs*. treatment): 94.9% *vs*. 96.7% (*p* > 0.05).	[285]
RIF (formula)	Leukemia	Alternating treatments with chemotherapy and RIF (6.0–7.5 g per day, *p.o.* 30 days of a cycle)	Treatment: 62 (no control group)	3-, 5-, 7- and 10-year relapsefree survival rates: 68.41%, 48.15%, 38.89%, 18.52%, respectively. 3-year and 5 to 10-year survival rates: 88.52% and 86.88%.	[286]
RIF (formula)	Leukemia	RIF (3.75–9 g per day, *p.o.*)	Multiple cases reports over many years: *n* = 18 to 204/report	CR rate: 91.67%-100%.	[287–291]

TCM: Traditional Chinese Medicine; CR: complete remission; PR: partial remission; SD: stable disease; RIF: Realgar-Indigo naturalis formula; TACE transcatheter arterial chemoembolization; TUV: trans-umbilical-vein; *i.v.*: intravenous perfusion; *i.p.*: intraperitoneal injection; *p.o.*: oral administration.

## Abbreviations

APL: acute promyelocytic leukemia
CAM: chicken embryo chorioallantoic membrane
CCC: countercurrent chromatography
CD: cyclodextrins
CPP: copalyl diphosphate
CR: complete remission
CT: cryptotanshinone
DAD: diode array
DH-TI: dihydrotanshinone I
DMAPP: dimethylallyldiphosphate
DXP: 1-deoxy-d-xylulose-5-phosphate
DXR: 1-deoxy-dxylulose- 5-phosphate reductoisomerase
DXS: 1-deoxy-d-xylulose-5-phosphate synthase
ECM: extracellular matrix
ESI-IT-MS: electrospray ionization quadrupole ion trap mass spectrometry
FBS: fetal bovine serum
FPP: farnesyl pyrophosphate
GA-3P: glyceraldehyde-3-phosphate
GGPP: geranylgeranyldiphosphate
GPP: geranyldiphosphate
HPLC: high-performance liquid chromatography
HSCCC: high-speed counter-current chromatography
*i.m.*: intramuscular
*i.p.*: intraperitoneal
IPP: isopentenyldiphosphate
i.s.*i.p.*: in situ intestine perfusion
*i.v.*: intravenous
LC: liquid chromatography
MEP: 2-*C*-methyl-d-erythritol-4-phosphate
MVA: mevalonic acid
MVD: microvessel density
*p.o.*: oral adminstration
PR: partial remission
qTOF-MS: quadrupole time-of-flight mass spectrometry
RIF: Realgar-Indigo naturalis formula
s.c.: subcutaneous
SD: stable disease
SLN: solid lipid nanoparticles
STS: sodium tanshinone IIA sulfonate
TI: tanshinone I
TIIA: tanshinone IIA
TACE: transcatheter arterial chemoembolization
TCM: Traditional Chinese Medicine
TLC: thin-layer chromatography
